# Evolutionary Analysis of Six Gene Families Part of the Reactive Oxygen Species (ROS) Gene Network in Three *Brassicaceae* Species

**DOI:** 10.3390/ijms25031938

**Published:** 2024-02-05

**Authors:** Thomas Horst Berthelier, Sébastien Christophe Cabanac, Caroline Callot, Arnaud Bellec, Catherine Mathé, Elisabeth Jamet, Christophe Dunand

**Affiliations:** 1 Laboratoire de Recherche en Sciences Végétales, Université de Toulouse, CNRS, UPS, Toulouse INP, 31320 Auzeville-Tolosane, France; thomas.berthelier@univ-tlse3.fr (T.H.B.); sebastien.cabanac@univ-tlse3.fr (S.C.C.); catherine.mathe-dehais@univ-tlse3.fr (C.M.); 2 Centre National de Ressources Génomiques Végétales, INRAE, 31320 Auzeville-Tolosane, France; caroline.callot@inrae.fr (C.C.); arnaud.bellec@inrae.fr (A.B.)

**Keywords:** adaptation, α-dioxygenase, ascorbate peroxidase, Brassicaceae, catalase, class III peroxidase, hypoxia, NADPH oxidase, salt stress, superoxide dismutase

## Abstract

Climate change is expected to intensify the occurrence of abiotic stress in plants, such as hypoxia and salt stresses, leading to the production of reactive oxygen species (ROS), which need to be effectively managed by various oxido-reductases encoded by the so-called ROS gene network. Here, we studied six oxido-reductases families in three *Brassicaceae* species, *Arabidopsis thaliana* as well as *Nasturtium officinale* and *Eutrema salsugineum*, which are adapted to hypoxia and salt stress, respectively. Using available and new genomic data, we performed a phylogenomic analysis and compared RNA-seq data to study genomic and transcriptomic adaptations. This comprehensive approach allowed for the gaining of insights into the impact of the adaptation to saline or hypoxia conditions on genome organization (gene gains and losses) and transcriptional regulation. Notably, the comparison of the *N. officinale* and *E. salsugineum* genomes to that of *A. thaliana* highlighted changes in the distribution of ohnologs and homologs, particularly affecting class III peroxidase genes (*CIII Prxs*). These changes were specific to each gene, to gene families subjected to duplication events and to each species, suggesting distinct evolutionary responses. The analysis of transcriptomic data has allowed for the identification of genes related to stress responses in *A. thaliana*, and, conversely, to adaptation in *N. officinale* and *E. salsugineum*.

## 1. Introduction

The current climate change increases intense episodes of drought and floods also leading to higher salinity rates at the soil surface. In the case of flooding or high salt concentration, plants must withstand partial or total immersion, as well as osmotic stress leading to Reactive Oxygen Species (ROS) homeostatic variations involving oxidoreductases (ORs) [1,2].

To adapt to various biotic and abiotic stresses, plants have evolved very complex regulatory mechanisms that can modulate the cellular concentration of ROS, including superoxide anion (O_2_^λ−^), hydrogen peroxide (H_2_O_2_), and hydroxyl radical (HO^λ^). High levels of ROS are toxic and lead to oxidative damage. When their concentrations are controlled, ROS participate in some biological processes such as cell growth, programmed cell death, and signaling [3]. ROS homeostasis is determined by the interplay between ROS-producing and scavenging mechanisms controlled by haem and non-haem peroxidases, as well as other ORs that are part of the so-called ROS gene network [4]. These proteins belong to more than 100 classes and are encoded by multigenic families comprising two to more than one hundred members (https://peroxibase.toulouse.inrae.fr/, accessed on 30 November 2023). Members of this ROS gene network have been detected in many species [5], and some families such as superoxide dismutases (SODs) and peroxiredoxins have been found in all kingdoms. Regulation of ROS homeostasis by the ROS gene network is a major player in salinity and hypoxia acclimation and adaptation [6,7,8].

Six gene families belonging to this ROS gene network have been selected for this study. (i) The ascorbate peroxidases (APxs), which are encoded by small multigenic families (1–10 members), are only present in chloroplastic organisms and are highly conserved between species. They play a key role in H_2_O_2_ homeostasis [9]. (ii) The monofunctional (typical) catalases (Kats) are present in all aerobic organisms in which they transform H_2_O_2_ into H_2_O and oxygen [10]. The number of *Kat* genes can vary from one to three copies. (iii) The class III peroxidases (CIII Prxs), which are encoded by large multigenic families (2–150 members), are only found in *Viridiplantae* and their genes have been subjected to numerous duplication events, which can be species specific [11]. CIII Prxs are mainly predicted as cell wall proteins and participate in many different biological processes such as cell wall elongation and stiffening, or protection against pathogens [12,13]. (iv) The α-dioxygenases (DiOxs) belong to small families (1–2 members). Their genes are only found in plants and are rarely subjected to duplication [14]. They catalyze the initial step of the α-oxidation of polyunsaturated fatty acids. Their activation is part of the defense mechanisms induced to protect cells from oxidative stress [15]. Moreover, their enzymatic activity is part of the plant responses to saline stresses [16]. (v) The NADPH oxidases (RBOHs) are encoded by small multigenic families (1–10 members) highly conserved between species. They are transmembrane proteins located at the plasma membrane producing O_2_^λ−^ extracellularly [17]. (vi) SODs are encoded by small multigene families (1–10 members). They catalyze the dismutation of O_2_^λ−^ into H_2_O_2_. In plants, there are three SOD classes (FeSOD, MnSOD and CuZnSOD), and two SOD-related classes: SOD-like (SDL) and copper chaperone for SOD (CCS) [18].

*Brassicaceae* is a large plant family composed of 57 tribes, 349 genera and 4140 species [19]. It is highly studied for its fundamental, evolutionary and agronomical aspects. In the NCBI database (https://www.ncbi.nlm.nih.gov/genome/?term=Brassicaceae, accessed on 30 November 2023) [20], 96 genomes are available, as well as 237,803 Sequence Read Archives (SRAs), mainly from *Arabidopsis thaliana* (166112) and *Brassica napus* (21486). For this study, three *Brassicaceae* species have been chosen based on the following criteria: genomic sequences and transcriptomic data availability, taxonomic proximity, and capacity to adapt to hypoxia or salt stress. *A. thaliana* has been chosen as a “reference” species (not halophytic, not resistant to hypoxia), *Eutrema salsugineum* (formerly *Thellungiella halophila*) as a halophytic species, and *Nasturtium officinale* (watercress) as a semi-aquatic species adapted to hypoxia.

To better understand the mechanisms of plant adaptation to salt stress or hypoxia, we have performed two complementary approaches. On the one hand, we have performed a complete and exhaustive phylogenomic analysis to see whether the number of genes encoding each of the above-described OR families was correlated to the capacity of the plants to cope with salt stress or hypoxia. In this respect, to complement the available genomic sequence of *N. officinale*, we provide new genomic data resulting from the assembly of larger DNA fragments compared to a previous study [21]. As a tetraploid species, *N. officinale* was also used to study the pseudogenization process/rate of two gene copies (ohnologs) in the six *OR* gene families. On the other hand, taking advantage of the availability of transcriptomics data, we have looked at the changes in the level of expression of the selected *ORs* to see if the regulation of some of them is modified depending on the capacity of the plants to cope with salt stress or hypoxia.

## 2. Results and Discussion

### 2.1. A New Genome of N. officinale

When this work started, no genomic data were available for *N. officinale*. Commercially available seeds were sown, and the genomic DNA was extracted from young leaves of 17 d old plantlets. The quality of the DNA has been checked and most of the DNA fragments were larger than 90 kb (Figure S1). The HiFiasm de novo assembler, initially designed for PacBio HiFi reads, produced a complete primary sequence comprising 2667 scaffolds, with the largest one at almost 12 Mb and more than 50% larger than 1 Mb (N50) (Figure S2). The overall coverage of the genome was 57x The calculated assembly size of *N. officinale* (primary total length of 647 Mb) was twice as big as the heterozygous genome size of 395 Mb estimated with the jellyfish tool [22], consistent with the value of 377 Mb previously determined by flow cytometry [23]. The observed difference suggested that the sequenced DNA could correspond to an admixture of genotypes. In addition, a BUSCO of 98.8% was calculated for the primary assembly with the brassicales_odb10.2020-08-05 lineage as a reference. This result indicated a high completeness of the genome sequence. A duplicated BUSCO of 74.1% could also be calculated, which was consistent with the tetraploidy of *N. officinale* [24].

At the time that we had finalized the genomic sequence of *N. officinale*, another sequencing project was released [21]. This former sequencing was performed with Illumina short reads, resulting in a number of scaffolds four times higher than in our work (10793). The value of N50 was 94.2 kb and the number of contigs was 14,564 (https://www.ncbi.nlm.nih.gov/datasets/genome/GCA_900406445.1/, accessed on 6 November 2023). The overall sequence was 216.1 Mb in length. This size was smaller than that obtained with our sequencing data, consistent with the fact that our data corresponded to several populations, most probably three, with a major one named Pop 1 (Table S1). This was mostly due to the use of commercial seeds containing mixed populations. In addition, pairs of genes with a lower percentage of identity between them than with genes from different populations (90% versus 98%, respectively) have been considered as ohnologs and numbered A and B. Ohnologs are actually paralogs originating from a whole-genome duplication event [25]. The BUSCO and the coverage of the former genome were comparable with those of the genome we sequenced, with values of 98.3% and 59.0 x, respectively. As an example, only three sequences (*NoffAPx02-1A*, *NoffPrx02-3B* and *NoffRboh[P]07-1B*) were not found in this previous genome (Table S1).

As mentioned above, we could conclude that the new genomic data provided by this study correspond to three populations of plants with a major one (Pop 1 in Tables S1 and S2). The availability of the former genomic sequence of *N. officinale* allowed us to clarify the distribution of the ohnolog and homolog genes between these populations (Tables S1 and S2). The complementary analyses of the two genomes show the presence of pairs of ohnologs and pseudogenes, as well as the gain and loss of orthologs in the gene families of interest (see Section 2.2). On the other hand, our genome with much longer scaffolds allowed for the determination of more precise gene distribution in the genome in the case of genes located in close proximity (Table S1). Indeed, the 189 genes identified in *N. officinale* in this study have been detected in 81 scaffolds in the case of our assembly vs. 159 in the case of the former genome. For example, the A copies of *NoffPrx69*, *70*, *71*, *72*, *73* (our work) are located on the same scaffold (ptg000119), whereas the corresponding genes of the previous genome are located on three different scaffolds (186 g, 3 g and 655 g) (Table S1). A similar situation was found for the B copies of the same genes. The localization of these five *Prx* genes on a single scaffold reflects the distribution of the ortholog genes in the *A. thaliana* genome.

The next step of this study was to perform a comparative phylogenetic analysis of six families of genes encoding proteins of the ROS network in three *Brassicaceae* adapted to different environmental conditions, *N. officinale*, *E. salsugineum* and *A. thaliana*.

### 2.2. Gene Duplications, Gains and Losses

*A. thaliana* and *N. officinale* belong to the *Camelineae* and *Cardamineae* tribes, respectively, which have diverged 20–30 MYA [26,27]. *E. salsugineum*, which belongs to the *Eutremeae* tribe, has diverged earlier, i.e., 24–35 MYA. *A. thaliana* and *E. salsugineum* are diploid species. It was recently shown that *E. salsugineum* did not experience an independent whole genome duplication event [28]. Conversely, *N. officinale* is a tetraploid. However, since this genome duplication event, some ohnologs could have been lost or pseudogenized.

In *E. salsugineum*, 10 *APxs*, 3 *Kats*, 74 *CIII Prxs*, 2 *DiOxs*, 11 *RBOHs* and 9 *SODs* have been found, including only eight pseudogenes for *CIII Prxs* (Table 1 and Table S2). The orthogroup analysis showed that only three *CIII Prxs* orthologs have been lost compared to *A. thaliana*, meaning that 15.9% of the orthologs of the *CIII Prxs* have been lost or are in a process of pseudogenization in *E. salsugineum*. No loss was observed for the five other gene families studied.

In *N. officinale*, 14 *APxs*, 5 *Kats*, 128 *CIII Prxs*, 3 *DiOxs*, 19 *RBOHs* and 18 *SODs* have been found, including 2, no, 18, no, 4 and 1 pseudogenes, respectively (Table 1 and Table S2). The orthogroup analysis showed that 2, 1, 16, 1, 1 and no orthologs, respectively, have been lost compared to *A. thaliana*, meaning that 23 to 25% of the ohnologs of five out of the six *OR* families of interest have been lost or are on the way to be lost by pseudogenization in *N. officinale*. Most of the gain or loss events are not common to *N. officinale* and *E. salsugineum* consistent with their distinct tribal classification: only 24% of the events observed in *N. officinale* also occurred in *E. salsugineum* (Table S2). However, some events are common (*Prx04*, *16*, *17*, *38*, *44* and *58*) and these events could be associated with a gene-specific selection pressure.

Two *CIII Prx* genes (*Prx16* and *22*) have been detected in *Camelineae* and *Cardamineae* while they are missing in *Eutremeae* (not found in *E. salsugineum* and *Eutrema halophilum*). The four *CIII Prx* genes (*Prx08*, *14*, *63* and *68*), which are missing in *N. officinale* and *E. salsugineum*, have been gained in *A. thaliana* through recent tandem, segmental or chromosomal duplications [29]. The chronology of these events is confirmed by the presence of *Prx63* and *Prx68* only in *A. thaliana* and in *Arabidopsis* species (e.g., *Arabidopsis lyrata*) and their lack in the other *Brassicaceae* tribes (e.g., *Prx63* and *68* are not detected in *Capsella rubella* and *Brassica napus*). Since the five other gene families (*APxs*, *Kats*, *DiOxs*, *RBOHs* and *SODs*) are much smaller than the *CIII Prx* families, they could be less subjected to the gain or loss of genes or pseudogenization events. Alternatively, the higher evolution rate of *CIII Prxs* could also be related to the diversity of their functions vs. the more specific functions of the five other gene families.

Independently of the sequence, the percentages of identity between the protein sequences of a given ortholog between the three species are very similar, with an average value of 85.7 ± 7.4% (Table S2). In addition, the percentages of identity are in the same range between (1) the orthologs, on the one hand, and (2) the ohnologs on the other hand, with an average value of 89.0% ± 10.3% for the latter, meaning that the selective pressure is sequence dependent, but similar at the intra- or inter-species levels. The Prx35 sequences are among the less conserved between species exhibiting 88.0% identity between ohnologs and 87.0% identity between orthologs (*N. officinale* vs. *A. thaliana*). However, they show 93.0% identity intra-tribe (*i.e.,* between *Camelineae*), as shown by a BLAST query using the Redoxibase (https://peroxibase.toulouse.inrae.fr/; accessed on 30 November 2023). Conversely, the Prx42 protein sequences are highly conserved between the three species exhibiting 98.0% identity between the *N. officinale* ohnologs, 97.0% identity between *A. thaliana* and *N. officinale* orthologs, and 97.0–98.0% with the protein sequences of their orthologs from other *Brassicaceae* tribes (*Arabideae*, *Brassiceae*, *Thlaspideae*). This could be related to the fact that Prx42 have crucial functions. Indeed, we have not yet been able to isolate *prx42* mutants (*F. Passardi* and *C. Dunand*, unpublished work).

The Ka/Ks ratio reflects the molecular evolution rate of a gene family. The ratio between the non-synonymous (Ka) and synonymous (Ks) substitution rates can be superior, inferior or equal to 1, indicating positive, purifying, or neutral selection, respectively [30]. The Ka/Ks ratios were calculated independently for each *OR* gene family (Figure S3). All of them were smaller than 1 (mean values between 0.03 and 0.19). It means that the mutations detected between orthologs and ohnologs are mostly synonymous, indicating that the evolution of the different *OR* families was driven by purifying selection pressure. This is probably related to the fact that the regulation of ROS homeostasis is highly critical, thus leading to low rates of evolution in the *OR* family genes. In addition, the percentage of identity is lower when one ortholog or one ohnolog is a pseudogene. These two results confirmed the hypothesis that the divergence between sequences (orthologs and ohnologs) is sequence dependent, thus possibly related to selective pressure. A similar evolutive scenario has already been described for the non-specific lipid transfer protein gene family in *Nicotiana tabacum* with a high percentage of identity between homologous sequences of three *Nicotiana* sp. and very low Ka/Ks ratios [31].

### 2.3. Differentially Expressed OR Genes upon Salt Stress

The differential expression of the *OR* genes of interest has then been analyzed upon salt stress in *A. thaliana* and *E. salsugineum*. Two experiments have been selected: (i) *A. thaliana* seedlings have been submitted to salt stress (50 mM NaCl) for 10 d in in vitro cultures [32], and (ii) 6-week-old *E. salsugineum* seedlings have been irrigated over 24 h with a 300 mM NaCl solution [33]. Both RNA-seq analyses were performed on roots.

**Figure 1:**
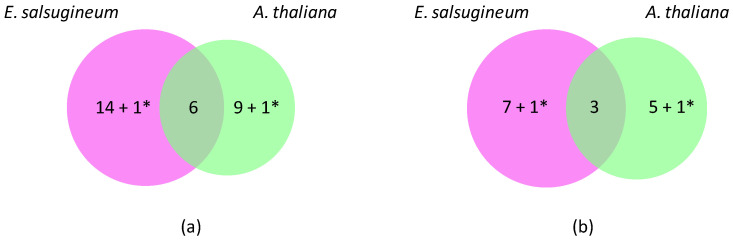
Differentially expressed *OR* genes in *A. thaliana* and *E. salsugineum* under salt stress. (**a**) Venn diagram showing specific (25) and common (6) down-regulated genes. (**b**) Venn diagram showing specific (14) and common (3) up-regulated genes. *A. thaliana* has thus been chosen as a reference to calculate the number of differentially expressed genes. * corresponds to genes with opposite regulations (*Prx10* and *APx01*).

Under these conditions, 46 *OR* genes with a significant FC (fold change) value have been detected (Figure 1). Eight *CIII Prxs* (two are up-regulated and six are down-regulated) and one *Kat* show a change in their expression level in a similar way in both plants (Table S3). Conversely, one *CIII Prx* is up-regulated in *A. thaliana* (*AtPrx10*), whereas its ortholog is down-regulated in *E. salsugineum* (*TsPrx10*), and one *APx* is down-regulated in *A. thaliana* (*AtAPx01*), whereas its ortholog is up-regulated in *E. salsugineum* (*TsAPx01*) (Table 2).

A total of 21 genes were specifically regulated in *E. salsugineum*, among which 6 were up-regulated and 15 down-regulated (Table 3). Among them, there are 15 *CIII Prxs*, 2 *APxs*, 2 *DiOxs* and 2 *SODs*. On the other hand, 14 genes were specifically regulated in *A. thaliana*, 5 were up-regulated and 9 were down-regulated (Table 3). Among them, there are nine *CIII Prxs*, two *RBOHs*, one *Kat* and three *SODs*. Some of the encoded proteins were found to be up- or down-accumulated by quantitative proteomics in *E. salsugineum* 4-week-old plants subjected to a 24 h watering with a 300 mM NaCl solution [34]. Some of these proteomics data show discrepancies with transcriptomics data, e.g., *TsAPx02*, *TsPrx21*, *TsKat02* and *TsCSD01* in Table 3 (see also [33,34]). This kind of difference has been described many times (for a review, see [35]).

*AtPrx01*, *AtPrx44* and *AtPrx73* are repressed by salt stress in the roots of both species (Table S3). These genes are mainly expressed in roots and control root hair length in *A. thaliana* [37]. *AtPrx34*, which is required for root elongation [38], is repressed in *A. thaliana*, but not differentially expressed in *E. salsugineum*. The observed modifications of *CIII Prx* expression are thus with a reduction of root growth in *A. thaliana* upon salt stress. Indeed, the salt stress response is a complex one in roots with a first phase of growth inhibition, followed by a second one of growth recovery [39]. In *E. salsugineum*, it was shown that salt stress results in an increased oxidation level of 159 Cys residues within 107 different proteins including proteins of the ROS network (TsPrx30, 49 and 71; TsKat02; TsCSD01). Interestingly, the *CIII Prx* homologous to *TsPrx03*, *07* and *56* are down-regulated in *Mesembryanthemum crystallinum*, a plant highly tolerant to salinity, after exposure to salt stress like in *E. salsugineum*, suggesting a role for these genes in adaptation to salinity [40].

In addition, none of the genes duplicated in *E. salsugineum* (*TsPrx22*, *33* and *38*; *APx03*; *Rboh07*) or of the *A. thaliana* genes corresponding to genes lost in *E. salsugineum* (*AtPrx22*, *33* and *38*) have a modified level of expression upon salt stress, suggesting that they play no critical role for the adaptation to salt stress (Table S2).

### 2.4. Differentially Expressed OR Genes upon Hypoxia

Another pair of plants has been selected to study the differential expression of the *OR* genes of interest upon hypoxia: *A. thaliana*, which is sensitive to hypoxia, and *N. officinale*, which is tolerant. The *A. thaliana* data originate from a comprehensive meta-study constituted by 29 RNA-seq datasets selected through a keyword search [41]. Instead of providing FC values, these datasets offer a HN-score, reflecting the trend of a gene being up- or down-regulated across all the experiments included in the study. These scores enabled us to compare the way in which a given gene is regulated, although they do not provide information on the extent of differential expression between control and treated plants. For *N. officinale*, the hypoxia stress was induced by submerging plants at the five or six-leaf stage in water for 24 h and the RNA-seq experiments were performed on the stems [42].

**Figure 2:**
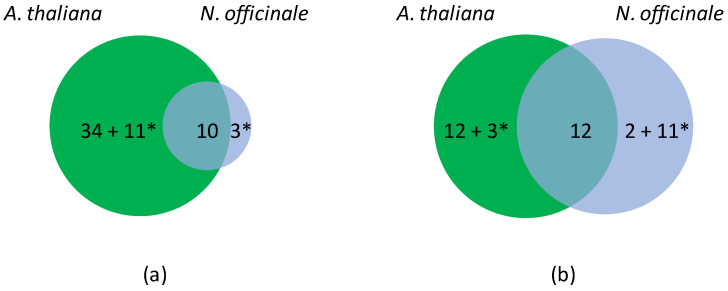
Differentially expressed *OR* genes in *A. thaliana* and *N. officinale* upon hypoxia. (**a**) Venn diagram showing unique (48) and common (10) down-regulated genes. (**b**) Venn diagram showing unique (28) and common (12) up-regulated genes. Note that the experimental data do not allow distinguishing ohnologs in *N. officinale*. *A. thaliana* has thus been chosen as a reference to calculate the number of differentially expressed genes. * corresponds to genes with contrasted regulations.

Upon hypoxia, 84 genes for which a significant FC value or a HN-score different from zero were found, as well as 28 genes of *N. officinale* for which no transcripts were detected (Table S3). Altogether, 36 genes were regulated in *A. thaliana* and *N. officinale* upon hypoxia, among which 10 and 12 were down- or up-regulated in both plants, respectively (Figure 2). Eleven genes were down-regulated in *A. thaliana*, but up-regulated in *N. officinale*. In contrast, three genes were up-regulated in *A. thaliana* but down-regulated in *N. officinale* (*APx02*, *Rboh04* and *FSD01*) (Table 4). In addition, 34 genes were down-regulated and 12 up-regulated in *A. thaliana* upon hypoxia, whereas only 2 genes were specifically up-regulated in *N. officinale*.

As in the case of the salt stress, it was possible to identify genes specifically regulated in *A. thaliana* upon hypoxia (Table 5). Thirty-five genes were down-regulated, among which included 23 *CIII Prxs*, 3 *APxs*, 1 *DiOx*, 1 *Rboh*, 2 *Kats* and 4 *SODs*. Fifteen genes were up-regulated among which nine were *Prxs* and three were *Rbohs*. Conversely, only two genes were specifically up-regulated in *N. officinale*: *Prx38* and *Rboh05*.

*AtPrx07*, *AtPrx44* and *AtPrx73* were found to be repressed in *A. thaliana* and not expressed in *N. officinale* (Table 5). *AtPrx44* and *AtPrx73* control root hair length in *A. thaliana* as shown by the phenotyping of mutants impaired in these genes and of over-expressors [37]. Yet, hypoxia inhibits root apical meristem (RAM) activity but increases root hair density and length [43].

*AtRboh04/AtRBOHD* was found to be up-regulated in many studies devoted to the analysis of the response of *A. thaliana* to hypoxia (HN-score of 26 out of the 29 analyzed sets of data), whereas it is weakly repressed in *N. officinale* (Table S3). This is consistent with the fact that the *AtRboh04*-mediated ROS burst induces genes required for hypoxia acclimation [44,45,46]. *AtRboh04* expression is detected in all organs, and AtRBOH04 could be the source of H_2_O_2_ in both roots and shoots during hypoxia.

As previously shown [47], *AtPrx04*, *AtPrx05* and *AtPrx28* are induced in *A. thaliana* upon hypoxia, but they are not differentially expressed in *N. officinale* (Table 5). As for *E. salsugineum* upon salt stress, none of the three duplicated genes in *N. officinale* is induced upon hypoxia (*NoffPrx17-2B*, *44-2A* and *44-2B*), meaning that their role in the adaptation to hypoxia is not critical. In the same way, the loss of several gene copies does not prevent *N. officinale* from being adapted to hypoxia (*NoffPrx04-1A*, *05-1B*, *30-1B*, *32-1B*, *46-1A*, *58-1B*, and *61-1B*; *NoffDiOx01-1B*; *NoffRboh07-1A*). Their orthologs in *A. thaliana* undergo changes in their regulation being either up- or down-regulated, thus suggesting a role in the response to hypoxia.

The perception of both saline stress and hypoxia first occurs at the root level when plants are moderately overflooded, but developmental effects, such as growth inhibition, can be also observed in the aerial part of the plants. Changes in gene expression in response to these stresses can therefore be rapidly detected after perception in the root, but also in the shoot following signal transduction [36].

About one third of the *A. thaliana OR* genes differentially regulated upon hypoxia or salinity were actually responding to both stresses, among which were 2 *APxs,* 1 *Kat*, 17 *Prxs*, 4 *SODs*, and 2 *Rbohs*. *AtPrx10* was identified as one of those genes and was previously shown to be involved in adventitious root formation, its mutant being impaired in root regeneration from leaf explants [48]. These genes could be part of a more general answer to abiotic stresses. None of them correspond to genes lost or gained in either genome. In addition, *AtPrx62* and its *N. officinale* ortholog were both induced during hypoxia (Table S3). Now, *AtPrx62* promotes root hair growth at low temperatures [49], which suggests that *AtPrx62* could be associated with root growth regulation in response to different stresses and induced even in tolerant species.

The number of genes belonging to the six *OR* gene families expressed in the adapted species or in the sensitive one and differentially regulated upon salt stress is lower than upon hypoxia. This is probably because many more experiments have been included in the latter case. However, for each stress, it was possible to identify genes specifically regulated either in *A. thaliana* or in the tolerant species and genes regulated in both cases.

## 3. Materials and Methods

### 3.1. Growth of N. officinale

*N. officinale* seeds (NAST-OFFI, Semences du Puy, Le Puy-en-Velay, France) were sown directly on water-saturated soil and grown with 50% humidity, at 21 °C upon a 13 h light/11 h dark cycle. The leaves were collected after 17 d, frozen in liquid nitrogen and stored at −80 °C, prior to DNA extraction.

### 3.2. HMW DNA Extraction

DNA was isolated from young leaves using a Genomic-tips 500/G kit (Cat No./ID: 10262, QIAGEN, Courtabœuf, France) following the tissue protocol extraction. Briefly, 2 g of young leaf material was ground in liquid nitrogen with a mortar and pestle. After 3 h of lysis at 50 °C and one centrifugation step, DNA was immobilized on the provided column. After several washing steps, DNA was eluted from the column, then desalted and concentrated by alcohol precipitation. The DNA was resuspended in the EB buffer. The DNA amount was assessed using a NanoDrop One spectrophotometer (Thermo Fisher Scientific, Illkirch-Graffenstaden, France) and a Qbit 3 Fluorometer using the Qbit dsDNA BR assay (Invitrogen, Illkirch-Graffenstaden, France). The size of the DNA fragments was determined using the FemtoPulse system (Agilent, Santa Clara, CA, USA) (Figure S1).

### 3.3. N. officinale Genome Sequencing: HiFi PacBio Library Preparation

According to the manual ‘Procedure & Checklist—Preparing HiFi SMRTbell Libraries using the SMRTbell Express Template Prep Kit 2.0′ (PN 101-853-100, Pacific Biosciences of California, San Diego, CA, USA), the HiFi libraries were prepared with 10 µg DNA sheared by using the Megaruptor 1 system (Diagenode, San Diego, CA, USA) to obtain a 15–20 kb average size. Size-selected libraries were sequenced on the PacBio Sequel II system in CCS mode for 30 h (Pacific Biosciences of California). To assess the quality and the completeness of the genomes, we performed BUSCO (version 5.4.4) analysis with the brassicales_odb10.2020-08-05 lineage as a reference [50].

### 3.4. Genome Annotation

We have annotated the newly sequenced *N. officinale* genome and the one deposited at NCBI (http://www.ncbi.nlm.nih.gov/datasets/genome/GCA_900406445.1/, accessed on 7 February 2023, [21]) with Braker3 [51]. We have also performed a functional annotation with InterProScan [52]. The v1.0 version of the genomic sequence of *E. salsugineum* (Esalsugineum_173_v1.fa) and the corresponding annotation file (Thalophila_173_gene.gff) were downloaded from Phytozome (http://phytozome-next.jgi.doe.gov/info/Esalsugineum_v1_0, accessed on 11 April 2023) [53]. From the perspective of more exhaustive evolutionary analysis, the prediction of pseudogene sequences was performed with P-GRe [54] in order to detect potential gene losses (pseudogenization).

Codon-based alignments of the coding regions were performed with MACSE v2 [55] for each orthogroup in each *OR* family, with default parameters, except the gap end penalty set to 8. Ka, Ks and subsequent Ka/Ks ratios were calculated from these alignments for each pair of sequences using the method of model averaging (MA) implemented in the KaKs_calculator 3.0 [56].

### 3.5. Phylogeny

Six gene families belonging to this ROS gene network have been extracted from the annotated genome using their PFAM ID. These families have been selected based on the two following criteria: the number of gene copies (possibly subjected to duplications), and the level of sequence conservation (possibly subjected to selective pressure). To precisely define the presence or absence of sequences in comparison with *A. thaliana*, the orthology and ohnology relationships were analyzed. All the protein sequences annotated and used in this study have been made available in the Redoxibase (https://peroxibase.toulouse.inrae.fr, accessed on 30 November 2023) [57]). Multiple alignments of the sequences were made with ClustalW [58]. Alignment curation and tree construction (PhyML) were made using NGPhylogeny.fr (https://ngphylogeny.fr/, accessed on 2 October 2023) [59]. In parallel, the orthogroups were inferred via the OrthoFinder software [60].

### 3.6. RNA-Seq Data

Three sets of RNA-seq data and one meta-analysis of RNA-seq have been used for this study. Plants were submitted to (i) salt stress for *A. thaliana* and *E. salsugineum* [32,33] or (ii) hypoxia stress for *A. thaliana* and *N. officinale* [41,42]. The details of the experimental setups are recalled as follows.

For salt stress, expression data for *A. thaliana* (Col-0 ecotype) [32] were obtained from roots grown in vitro for 10 d in half-strength Murashige and Skoog basal medium (1/2 MS) with and without 50 mM NaCl. For *E. salsugineum* (Shandong ecotype), expression data were collected from roots of plants germinated on 1/2 MS, transferred to soil after 7 d for a 5-week-growth period, and subjected to either 300 mM NaCl solution (salt treated) or water (control) irrigation for 24 h before sampling. These two NaCl concentrations have been described as stressful [61] but not toxic for the two species [32,34].

In the case of hypoxia, metadata for *A. thaliana* were sourced from 29 RNA-seq datasets identified through a keyword search in the Gene Expression Omnibus (GEO) [62]. Most samples consisted of whole seedlings or aerial organs. For *N. officinale*, expression data were obtained from plant stems that were initially germinated in sterile soil, grown until they reached the 5–6 leaf stage, and then sampled after 24 h of submersion to induce hypoxia.

### 3.7. Pipeline for the OR Families Expression Analysis

In hypoxia conditions for *A. thaliana* (meta-analysis), differential expressions have been calculated in the form of an HN-score (hypoxia–normoxia score), as computed by the authors [41]. So, no associated statistical values were provided. For the other differential analyses, FCs with a Benjamini–Hochberg adjusted *p*-value (FDR or false discovery rate) have been employed. Differential expressions utilized for *N. officinale* [42] and *A. thaliana* [32] under saline conditions were directly derived from the published data.

Regarding *E. salsugineum*, RNAseq libraries (SRR6837742, SRR6837743, SRR14804236, SRR14804235, available at NCBI) were used for conducting a differential analysis using the nf-core/rnaseq pipeline version 3.10.1 in Nextflow v22.12.0 [63] with default parameters [64,65]. Next, the counts were normalized using the Trimmed Mean of M-values (TMM) method from the EdgeR package [66,67]. Subsequently, a selection of expressed genes was performed, using a threshold of expression higher than 0.1 cpm (count per million) across all replicates. Following a second TMM normalization on the expressed genes, an analysis was conducted using the EdgeR package. The expression of the log_2_FC threshold was set to 0 to distinguish the expressed genes (even if not differentially expressed) from genes with undetectable levels of expression.

To select the transcripts corresponding to the six investigated *OR* gene families, a BLAST analysis was carried out with the gene sequences annotated as described above.

## 4. Conclusions

This work has allowed for the expert annotation of 110 *OR* genes belonging to six different families in the diploid *E. salsugineum* and 188 in the tetraploid *N. officinale*. They were compared to the 108 already-annotated *OR* genes of *A. thaliana*. We could show that most of the orthologous sequences are conserved between the three species, which probably diverged 30 MYA, thus supporting the idea that *OR* families have critical functions for ROS homeostasis. As for other plants, polyploidy could have been an asset for the adaptation and evolution of *N. officinale* [68]. Also, we have observed that less than 30% of the ohnologs of *N. officinale* have been lost or pseudogenized, suggesting that the conservation of the two copies brought sufficient advantages (such as sub-functionalization or dose effect) to bypass the loss of duplicated genes [69].

Global gene expression analyses showed genes similarly regulated in the species subjected to comparable stresses. However, we also observed genes only regulated by stress in *A. thaliana*, which is sensitive to both stresses. Conversely, there were genes only regulated in the halophytic *E. salsugineum* upon salt stress or in the hypoxia-tolerant *N. officinale* upon hypoxia. In addition, among the six *OR* classes studied, *CIII Prx* genes represent two thirds of the genes analyzed and have been largely described as stress markers [12]. Their roles seem to be critical, especially upon hypoxia. It is also important to recall that the six *OR* classes analyzed in this work are members of the so-called ROS gene network, which contains dozens of other *OR* classes participating in ROS homeostasis during regular metabolism but also following stressing events. The *OR* genes highlighted by this work could be targets for functional analyses to better understand their roles either in the acclimation to hypoxia or salinity or in the stress responses.

## Figures and Tables

**Table 1:** *APxs*, *Kats*, *CIII Prxs*, *DiOxs*, *RBOHs* and *SOD* genes have been annotated from the three *Brassicaceae* species (Table S2). Open reading frames with relevant predicted functional domains and pseudogenes (Ψ, corresponding to truncated amino acid sequence, nucleotidic sequences with in-frame stop codons, frameshifts or gaps) are indicated. Gene gains and losses have been determined using the *A. thaliana* orthologs as references. Only A and B genes of the major population of *N. officinale* have been considered for the calculations (Pop 1, Table S2).

Gene Family		*E. salsugineum*	*A. thaliana*	*N. officinale*
*APx*	Total gene number	10	8 + 1 Ψ	12 + 2 Ψ
	% Ψ	0	11.1	14.3
	Gene gain	2	0	0
	Gene loss	0	0	2
	% gene lost + Ψ	0	nd	25.0
*Kat*	Total gene number	3	3	5
	% Ψ	0	0	0
	Gene gain	0	0	0
	Gene loss	0	0	1
	% gene lost + Ψ	0	nd	16.6
*CIII Prx*	Total gene number	66 + 8 Ψ	73 + 2 Ψ	111 + 17 Ψ
	% Ψ	10.8	2.7	14.1
	Gene gain	8	5	5
	Gene loss	3	0	16
	% gene lost + Ψ	15.9	nd	24.5
*DiOx*	Total gene number	2	2	3
	% Ψ	0	0	0
	Gene gain	0	0	0
	Gene loss	0	0	1
	% gene lost + Ψ	0	nd	25.0
*RBOH*	Total gene number	11	10	15 + 4 Ψ
	% Ψ	0	0	21.0
	Gene gain	1	0	0
	Gene loss	0	0	1
	% gene lost + Ψ	0	nd	25.0
*SOD*	Total gene number	9	9	17 + 1 Ψ
	% Ψ	0	0	5.5
	Gene gain	0	0	0
	Gene loss	0	0	0
	% gene lost + Ψ	0	nd	5.5

**Table 2:** *OR* genes regulated in contrasted manner between *A. thaliana* and *E. salsugineum* upon salt stress. The gene nomenclature is that of the online database Redoxibase (https://peroxibase.toulouse.inrae.fr/; accessed on 30 November 2023). Differential expression data are presented as log_2_(FC).

*E. salsugineum* Redoxibase ID	*E. salsugineum* log_2_(FC)	*A. thaliana* log_2_(FC)	*A. thaliana* Redoxibase ID
*TsPrx10/Thhalv10011619*	−3.01	15.87	*AtPrx10/At1g49570*
*TsAPx01/Thhalv10008402m*	0.67	−1.41	*AtAPx01/At1g07890*

**Table 3:** *APxs*, *Kats*, *CIII Prxs*, *DiOxs*, *RBOHs* and *SODs* differentially expressed upon salt stress in *E. salsugineum* and *A. thaliana*. The gene nomenclature is that of the online database Redoxibase (https://peroxibase.toulouse.inrae.fr/; accessed on 30 November 2023). Differential expression data are presented as log_2_(FC). ‘No transcript’ indicates that no transcripts were detected, and ‘FDR > 0.05’ that the calculated FDR is greater than 0.05 (not statistically significant). (+) and (−) in the second [34] and third [36] columns indicate that the proteins were found to be up- or down-accumulated upon salt stress, respectively.

*E. salsugineum* Redoxibase ID	*E. salsugineum* log2(FC)	*A. thaliana* log2(FC)	*A. thaliana* Redoxibase ID
*TsPrx02/Thhalv10008157*	−2.88	FDR > 0.05	*AtPrx02/At1g05250*
*TsPrx03/Thhalv10008189*	−0.69	FDR > 0.05	*AtPrx03/At1g05260*
*TsPrx11/Thhalv10018830*	0.97	FDR > 0.05	*AtPrx11/At1g68850*
*TsPrx12/Thhalv10018753*	−2.07	FDR > 0.05	*AtPrx12/At1g71695*
*TsPrx25/Thhalv10016924*	−0.89	FDR > 0.05	*AtPrx25/At2g41480*
*TsPrx27/Thhalv10021152*	−1.96	FDR > 0.05	*AtPrx27/At3g01190*
*TsPrx28/Thhalv10021155*	−2.02	no FDR	*AtPrx28/At3g03670*
*TsPrx30/Thhalv10021123*	−0.85 (+)	FDR > 0.05	*AtPrx30/At3g21770*
*TsPrx39/Thhalv10028797*	−2.80	FDR > 0.05	*AtPrx39/At4g11290*
*TsPrx45/Thhalv10025699*	−1.65	FDR > 0.05	*AtPrx45/At4g30170*
*TsPrx56/Thhalv10014085*	−1.44	FDR > 0.05	*AtPrx56/At5g15180*
*TsPrx58-1/Thhalv10014083*	−2.15 (−)	FDR > 0.05	*AtPrx58/At5g19880*
*TsPrx59/Thhalv10014080*	−2.84	FDR > 0.05	*AtPrx59/At5g19890*
*TsPrx66/Thhalv10014117*	2.43	FDR > 0.05	*AtPrx66/At5g51890*
*TsPrx69/Thhalv10004557*	−0.62	FDR > 0.05	*AtPrx69/At5g64100*
*TsAPx-R/Thhalv10025686m*	0.96	FDR > 0.05	*AtAPx-R/At4g32320*
*TsAPx02/Thhalv10021381m*	1.87 (+)	FDR > 0.05	*AtAPx02/At3g09640*
*TsDiOx01/Thhalv10020279*	−3.33	FDR > 0.05	*AtDiOx01/At3g01420*
*TsDiOx02/Thhalv10018266*	0.97	FDR > 0.05	*AtDiOx02/At1g73680*
*TsFSD01-1A/Thhalv10026218*	1.11	FDR > 0.05	*AtFSD01/At4g25100*
*TsMSD01-1A/Thhalv10021449*	0.61	FDR > 0.05 (+)	*AtMSD01/At3g10920*
no sequence		−4.04	*AtPrx08/At1g34510*
*TsPrx21/Thhalv10016921*	FDR > 0.05 (−)	−1.74	*AtPrx21/At2g37130*
*TsPrx34/Thhalv10010494*	FDR > 0.05	−1.69 (+)	*AtPrx34/At3g49120*
*TsPrx35/Thhalv10010538*	FDR > 0.05	−3.61	*AtPrx35/At3g49960*
*TsPrx42/Thhalv10025680*	FDR > 0.05	−1.29	*AtPrx42/At4g21960*
*TsPrx50/Thhalv10025717*	no transcript	1.61	*AtPrx50/At4g37520*
*TsPrx54/Thhalv10013916*	no transcript	−1.97	*AtPrx54/At5g06730*
*TsPrx60/Thhalv10015253*	no transcript	−5.01	*AtPrx60/At5g22410*
*TsRboh03/Thhalv10012613*	FDR > 0.05	−1.46	*AtRboh03/At5g51060*
*TsRboh04/Thhalv10003619*	FDR > 0.05	−1.40	*AtRboh04/At5g47910*
*TsKat02/Thhalv10025015*	no transcript (+)	1.32	*AtKat02/At4g35090*
*TsCSD01/Thhalv10008927*	FDR > 0.05 (+)	1.80	*AtCSD01/At1g08830*
*TsCSD02/Thhalv10017094*	FDR > 0.05	1.77	*AtCSD02/At2g28190*
*TsCCS01/Thhalv10008232*	FDR > 0.05	1.92	*AtCCS01/At1g12520*

**Table 4:** *OR* genes regulated in contrasted manner between *A. thaliana* and *N. officinale* upon hypoxia. The gene nomenclature is that of the online database Redoxibase (https://peroxibase.toulouse.inrae.fr/; accessed on 30 November 2023). Differential expression data are presented as log_2_(FC).

*N. officinale* Redoxibase ID	*N. officinale* log_2_(FC)	*A. thaliana* HN-score	*A. thaliana* Redoxibase ID
*NoffPrx09-1B*	5.56	−3	*AtPrx09/At1g44970*
*NoffPrx16-1A*	3.18	−5	*AtPrx16/At2g18980*
*NoffPrx22-1A*	6.16	−1	*AtPrx22/At2g38380*
*NoffPrx23-1A*	6.36	−2	*AtPrx23/At2g38390*
*NoffPrx27-1A*	3.83	−2	*AtPrx27/At3g01190*
*NoffPrx45-1A*	2.87	−4	*AtPrx45/At4g30170*
*NoffPrx56-1A*	4.27	−2	*AtPrx56/At5g15180*
*NoffPrx64-1A*	1.92	−2	*AtPrx64/At5g42180*
*NoffPrx67-1A*	4.59	−2	*AtPrx67/At5g58390*
*NoffAPx02-1A*	−3.56	1	*AtAPx02/At3g09640*
*NoffRboh04-1A*	−0.40	26	*AtRboh04/At5g47910*
*NoffCCS01-1A*	1.97	−3	*AtCCS01/At1g12520*
*NoffCSD03-1A*	0.74	−1	*AtCSD03/At5g18100*
*NoffFSD01-1A*	−5.38	3	*AtFSD01/At4g25100*

**Table 5:** *APxs*, *Kats*, *CIII Prxs*, *DiOxs*, *RBOHs* and *SODs* differentially expressed upon hypoxia stress in *N. officinale* and *A. thaliana*. The gene nomenclature is that of the online database Redoxibase (https://peroxibase.toulouse.inrae.fr/; accessed on 30 November 2023). Differential expression data are presented as log_2_(FC). Differential expression data are presented in log_2_(FC) for *N. officinale* and HN-score for *A. thaliana* [42]. ‘No transcript’ indicates that no transcripts were detected, ‘FDR > 0.05’ that the calculated FDR was greater than 0.05 (not statistically significant), and [P] that no transcripts were detected because the gene was annotated as a pseudogene.

*N. officinale* Redoxibase ID	*N. officinale* log_2_(FC)	*A. thaliana* HN-score	*A. thaliana* Redoxibase ID
NoffPrx38-1A	2.42	0	*AtPrx38/At4g08780*
*NoffRboh05*	1.38	0	*AtRboh05/At1g19230*
*NoffPrx02-1A*	no transcript	−4	*AtPrx02/At1g05250*
*NoffPrx04-1B*	FDR > 0.05	15	*AtPrx04/At1g14540*
*NoffPrx05-1A*	no transcript	6	*AtPrx05/At1g14550*
*NoffPrx07-1A*	no transcript	−4	*AtPrx07/At1g30870*
no sequence		−13	*AtPrx08/At1g34510*
*NoffPrx11-1A*	no transcript	−2	*AtPrx11/At1g68850*
*NoffPrx12-1A*	FDR > 0.05	−9	*AtPrx12/At1g71695*
*NoffPrx15-1A*	no transcript	−3	*AtPrx15/At2g18150*
*NoffPrx17-1A*	FDR > 0.05	−1	*AtPrx17/At2g22420*
*NoffPrx21-1A*	FDR > 0.05	2	*AtPrx21/At2g37130*
no sequence		−5	*AtPrx24/At2g39040*
*NoffPrx25-1A*	no transcript	−11	*AtPrx25/At2g41480*
*NoffPrx28-1A*	no transcript	5	*AtPrx28/At3g03670*
*NoffPrx30-1A*	FDR > 0.05	−3	*AtPrx30/At3g21770*
*NoffPrx32-1A*	FDR > 0.05	−2	*AtPrx32/At3g32980*
*NoffPrx33-1A*	FDR > 0.05	−1	*AtPrx33/At3g49110*
*NoffPrx[P]34-1A*	[P]	5	*AtPrx34/At3g49120*
*NoffPrx35-1B*	no transcript	−6	*AtPrx35/At3g49960*
*NoffPrx39-1A*	FDR > 0.05	−2	*AtPrx39/At4g11290*
*NoffPrx40-1A*	FDR > 0.05	−1	*AtPrx40/At4g16270*
*NoffPrx44-1A*	no transcript	−3	*AtPrx44/At4g26010*
*NoffPrx46-1B*	FDR > 0.05	10	*AtPrx46/At4g31760*
*NoffPrx53-1A*	no transcript	−3	*AtPrx53/At5g06720*
*NoffPrx57-1A*	no transcript	−2	*AtPrx57/At5g17820*
*NoffPrx58-1A*	FDR > 0.05	2	*AtPrx58/At5g19880*
*NoffPrx59-1A*	no transcript	6	*AtPrx59/At5g19890*
*NoffPrx60-1A*	no transcript	−10	*AtPrx60/At5g22410*
*NoffPrx61-1A*	no transcript	6	*AtPrx61/At5g24070*
no sequence		−11	*AtPrx63/At5g40150*
*NoffPrx65-1A*	no transcript	−1	*AtPrx65/At5g47000*
*NoffPrx72-1A*	FDR > 0.05	−2	*AtPrx72/At5g66390*
*NoffPrx73-1A*	FDR > 0.05	−4	*AtPrx73/At5g67400*
*NoffAPx-R01-1A*	FDR > 0.05	−2	*AtAPx-R/At4g32320*
*NoffAPx03-1A*	FDR > 0.05	−1	*AtAPx03/At4g35000*
*NoffAPx06-1A*	FDR > 0.05	−5	*AtAPx06/At1g77490*
*NoffDiOx01-1A*	FDR > 0.05	−1	*AtDiOx01/At3g01420*
*NoffRboh03-1A*	no transcript	3	*AtRboh03/At5g51060*
*NoffRboh06-1A*	FDR > 0.05	1	*AtRboh06/At1g64060*
*NoffRboh07-1B*	FDR > 0.05	−1	*AtRboh07/At4g25090*
*NoffRboh09-1B*	no transcript	3	*AtRboh09/At4g11230*
*NoffKat01-1B*	FDR > 0.05	−2	*AtKat01/At1g20630*
*NoffKat02-1A*	FDR > 0.05	−4	*AtKat02/At4g35090*
*NoffCSD02-1A*	FDR > 0.05	−1	*AtCSD02/At2g28190*
*NoffFSD02-1A*	FDR > 0.05	−2	*AtFSD02/At5g51100*
*NoffFSD03-1A*	FDR > 0.05	−4	*AtFSD03/At5g23310*
*NoffMSD01-1A*	FDR > 0.05	−1	*AtMSD01/At3g10920*

## Data Availability

The *N. officinale* protein sequences translated from the genome available at NCBI (Kiefer. 2019) are available in the Redoxibase (https://peroxibase.toulouse.inrae.fr, accessed on 30 November 2023).

## Supplementary Materials

The following supporting information can be downloaded at: https://www.mdpi.com/article/10.3390/ijms25031938/s1.
