# Supplementary material for: Evolutionary Analysis of Six Gene Families Part of the Reactive Oxygen Species (ROS) Gene Network in Three Brassicaceae Species
Source: Int J Mol Sci. 2024 Feb 5;25(3):1938. doi: 10.3390/ijms25031938 (PMC10856686; doi:10.3390/ijms25031938)
Supplement: Supplementary file 1 [file ijms-25-01938-s001.zip › Berthelier_et_al_Supplementary Figures.pdf]

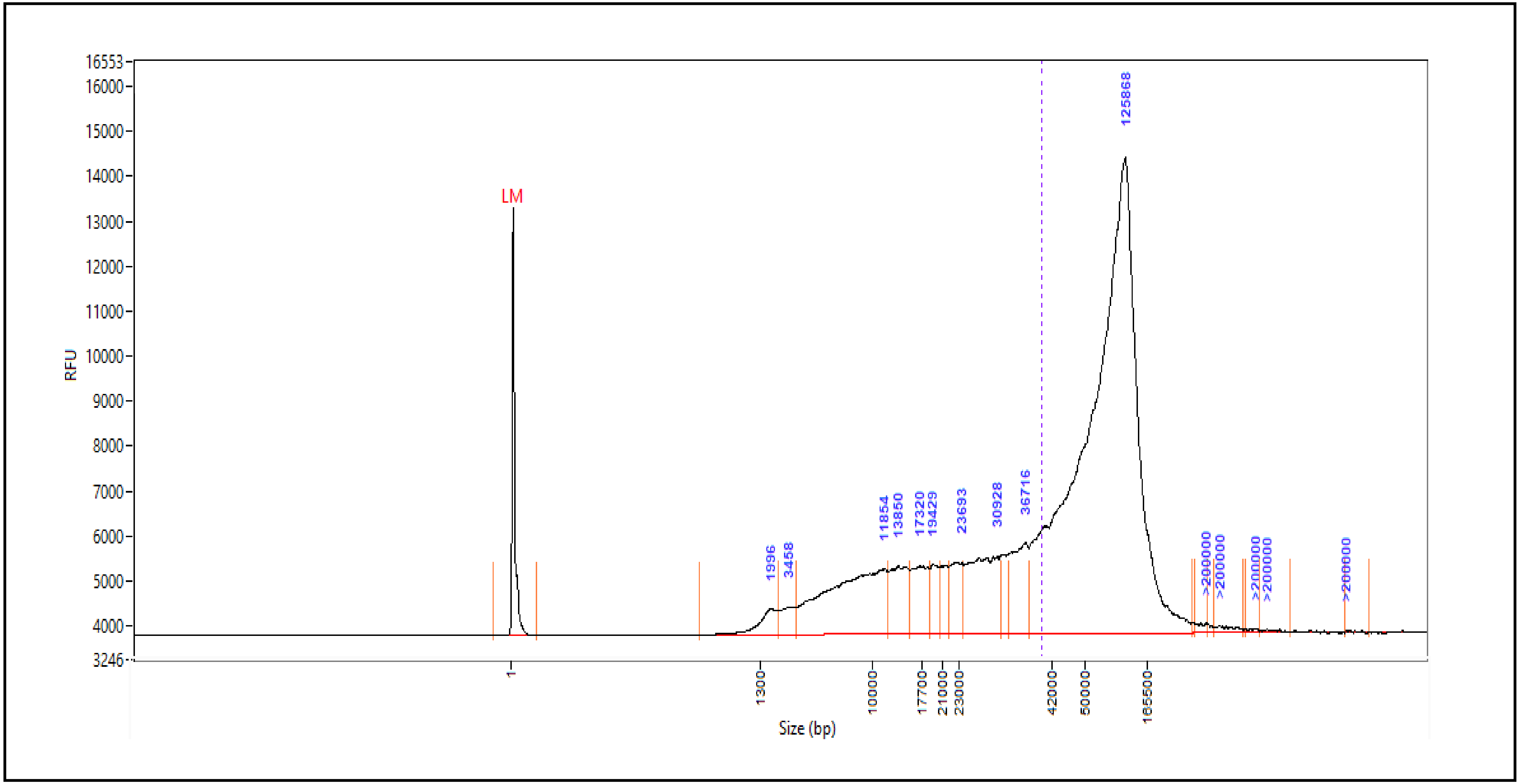

Figure S1. FemtoPulse® system (Agilent) electrophoregram of *Nasturtium officinale* High Molecular Weight genomic DNA (gDNA) extracted with the Qiagen Genomic Tip protocol (see Material and Methods for details). Most of the gDNA fragments are larger then 90 kb.

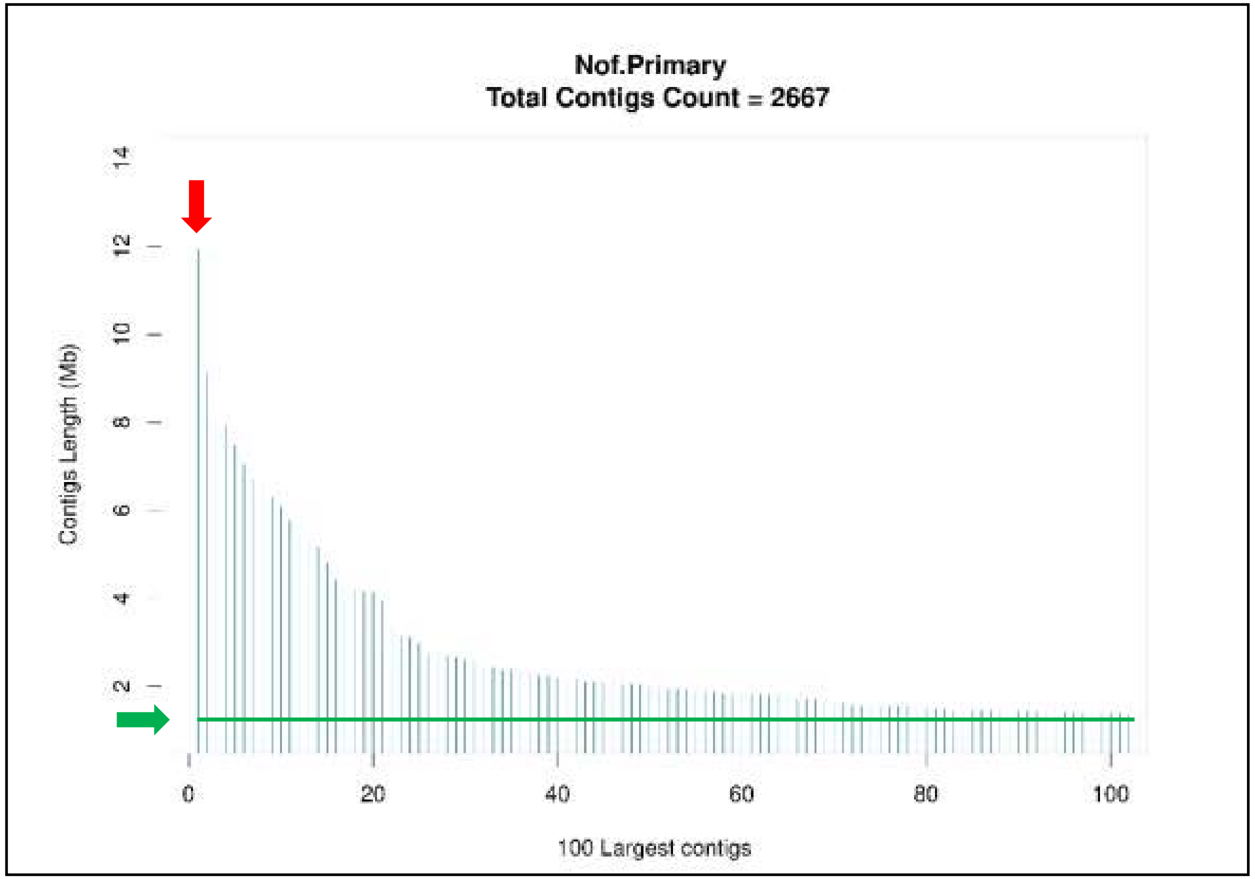

Figure S2. Distribution of contigs according to their length: the largest one is almost 12 Mb in length (red arrow) and 50% of them are larger than 1 Mb (green arrow).

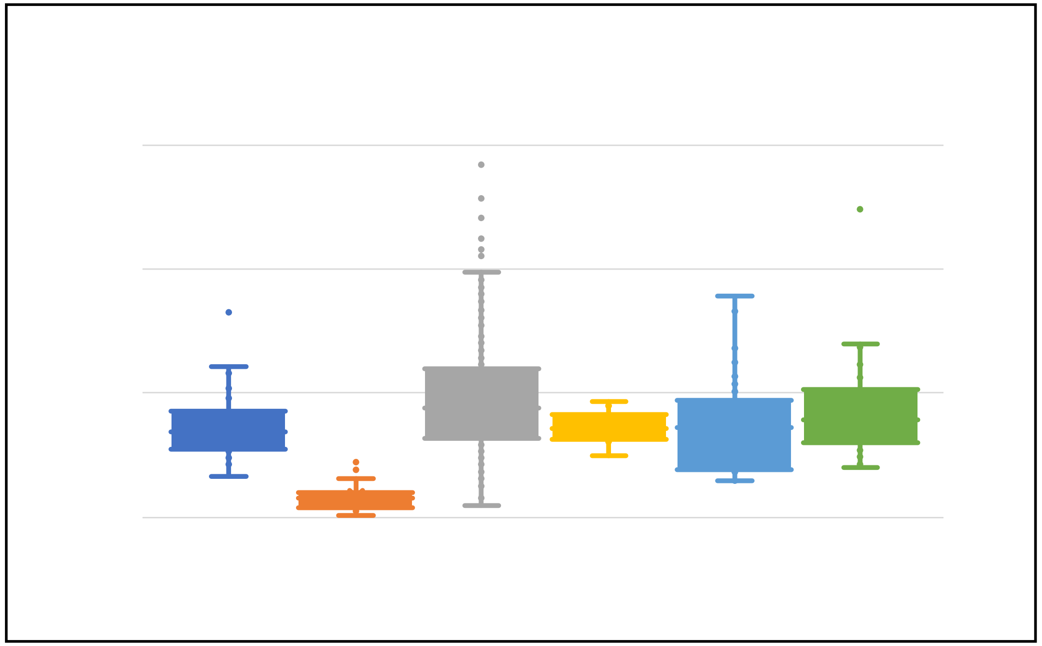

Figure S3. Box plots of Ka/Ks ratios calculated for each pair of orthologs within each *OR* family.
